# The role of hydrogen in heavy transport to operate within planetary boundaries†

**DOI:** 10.1039/d1se00790d

**Published:** 2021-07-30

**Authors:** Antonio Valente, Victor Tulus, Ángel Galán-Martín, Mark A. J. Huijbregts, Gonzalo Guillén-Gosálbez

**Affiliations:** Department of Chemistry and Applied Biosciences, Institute for Chemical and Bioengineering, ETH Zurich 8093 – Zurich Switzerland gonzalo.guillen.gosalbez@chem.ethz.ch; Department of Chemical, Environmental and Materials Engineering, Universidad de Jaén Campus Las Lagunillas s/n 23071 Jaén Spain; Center for Advanced Studies in Earth Sciences, Energy and Environment. Universidad de Jaén Campus Las Lagunillas s/n 23071 Jaén Spain; Department of Environmental Science, Institute for Water and Wetland Research, Radboud University Nijmegen The Netherlands

## Abstract

Green hydrogen, *i.e.*, produced from renewable resources, is attracting attention as an alternative fuel for the future of heavy road transport and long-distance driving. However, the benefits linked to zero pollution at the usage stage can be overturned when considering the upstream processes linked to the raw materials and energy requirements. To better understand the global environmental implications of fuelling heavy transport with hydrogen, we quantified the environmental impacts over the full life cycle of hydrogen use in the context of the Planetary Boundaries (PBs). The scenarios assessed cover hydrogen from biomass gasification (with and without carbon capture and storage [CCS]) and electrolysis powered by wind, solar, bioenergy with CCS, nuclear, and grid electricity. Our results show that the current diesel-based-heavy transport sector is unsustainable due to the transgression of the climate change-related PBs (exceeding standalone by two times the global climate-change budget). Hydrogen-fuelled heavy transport would reduce the global pressure on the climate change-related PBs helping the transport sector to stay within the safe operating space (*i.e.*, below one-third of the global ecological budget in all the scenarios analysed). However, the best scenarios in terms of climate change, which are biomass-based, would shift burdens to the biosphere integrity and nitrogen flow PBs. In contrast, burden shifting in the electrolytic scenarios would be negligible, with hydrogen from wind electricity emerging as an appealing technology despite attaining higher carbon emissions than the biomass routes.

## Abstract 2

Fueling trucks with green hydrogen shows a favorable environmental life-cycle performance with respect to conventional diesel. Hydrogen as an alternative fuel would help society to safely operate within the absolute ecological limits of our planet.

## Graphical abstract

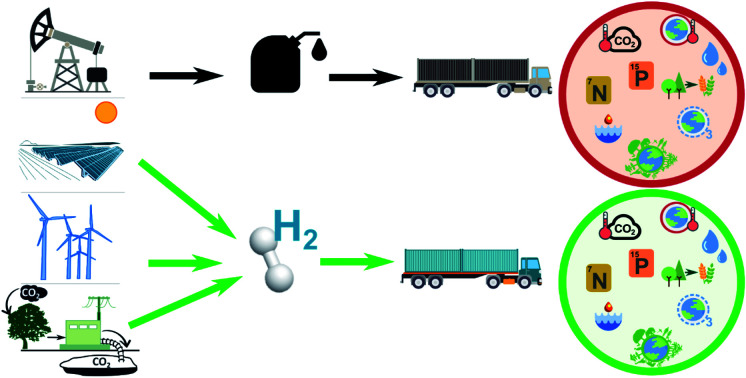

## Introduction

Achieving carbon neutrality by 2050 is one of the most challenging goals currently set by governments concerning environmental policy.^1^ Nowadays, the energy and transport sectors are the main driving forces of anthropogenic environmental pressure. In 2018, 81% of the world's primary energy supply was covered by fossil fuels.^2^ The transport sector, which alone accounts for one-fifth of the global primary energy demand, almost entirely relies on petroleum, *i.e.*, 96% of the energy demand.^2^ Moreover, the progressive growth in fuel consumption and an increase in the number of vehicles (especially in non-OECD countries) further stress the urgency of finding alternative transport options.^3^

In the quest towards sustainable mobility, many countries are introducing policies to promote battery electric vehicles (BEVs) that would substitute fossil-based internal combustion engine vehicles.^4^ Although BEVs are suitable alternatives for urban and micro-mobility, technical limitations such as high recharge duration and limited travel autonomy jeopardise the application of battery-based technologies as realistic solutions for heavy-duty vehicles.^5^ In this context, green hydrogen – *i.e.*, produced from renewable resources – is attracting attention as an alternative fuel that may play a vital role in the future of heavy road transport and long-distance driving in commercially available solutions.^4,6–9^ However, depending on the hydrogen production pathways, the benefits of zero pollution in the use phase can be overturned when considering the life-cycle impacts embodied in raw materials and energy inputs.^10^ For this reason, covering the entire life-cycle is key for a proper assessment of hydrogen as an alternative fuel.

Life Cycle Assessment (LCA) is recognised as a central tool to evaluate human activities' impacts from a life-cycle perspective^11,12^ and it is becoming essential to underpin evidence-based policies in the EU.^13^ LCA is traditionally used to identify environmental hotspots across value chains and benchmark the performance of a range of alternatives, often called scenarios, against a reference system or target. Different LCA studies analysed hydrogen production and use systems. Sternberg and Bardow^14^ explored energy storage alternatives (including renewable electrolytic hydrogen) as a potential power-to-fuel solution. The authors concluded that the direct use of hydrogen (injected into the natural gas grid or used in fuel cell vehicles) is more beneficial than its use to produce fuels (methane, methanol, or syngas) in terms of global warming and fossil depletion impact. The LCA study performed by Bargiacchi *et al.*^15^ concluded that natural gas from renewable hydrogen and biomass is environmentally better than its fossil analogue. Adopting also a life-cycle perspective, Abanades *et al.*^16^ investigated the use of renewable energy to either produce methanol or decarbonise the power mix. The authors found that the latter shows higher carbon reduction potential, yet both are suitable mitigation strategies. Focusing on the transport sector, the study carried out by Al-Qahtani *et al.*^17^ pointed out that the use of renewable hydrogen and CO_2_ captured from fossil power plants to produce a fuel blend (10% gasoline and 90% methanol) has environmental benefits compared to conventional gasoline. However, as in Abanades *et al.*,^16^ Al-Qahtani and co-workers^17^ concluded that, under the current scenario, the preferred option is to use renewable electricity to decarbonise the power system. Several authors agree on the suitability of hydrogen in fuel cell passenger vehicles as an alternative mobility option.^18–24^ In this regard, the review presented by Valente *et al.*^25^ found increasing scientific interest in assessing the environmental performance of hydrogen systems through LCA. In particular, the review pointed out that most LCA studies focused on the decarbonisation of the transport sector as a whole using hydrogen as an alternative fuel. However, specific studies addressing the life-cycle performance of heavy trucks fuelled with hydrogen are scarce. Among the few studies addressing this topic, Lee and colleagues^26^ compared the environmental life-cycle performances of conventional diesel-fuelled trucks with that of fuel cell hydrogen trucks in terms of fossil fuel consumption, GHG and polluting emissions in the US. Although in their study the authors considered a fossil feedstock-based route to produce hydrogen (namely, steam reforming of natural gas), they concluded that this solution would reduce the environmental pressure of heavy transport and that renewable hydrogen would be further beneficial under all the environmental aspects analysed. In another study, El Hannach *et al.*^27^ compared the environmental and techno-economic aspects of a fleet composed of 200 dual fuel trucks (fuelled with a gasoline-hydrogen blend with different hydrogen-to-diesel ratios) with those of a fleet of conventional diesel trucks. In their analysis, hydrogen was considered to be sourced from chemical facilities that normally produce hydrogen as a waste. The authors found a significant drop in GHG and other polluting emissions as well as operating costs.

Though widespread, most LCA studies fail to accurately contextualise an environmental profile in terms of absolute sustainability. Notably, LCA indicators are useful for comparing alternative products, including fuels, but because they lack impact thresholds that should not be exceeded globally, they cannot establish whether these are sustainable in absolute terms. Therefore, a more holistic approach connecting the LCA impacts of a system to its absolute performance at the planetary level is required to evaluate its absolute sustainability level. Neglecting these global impacts might lead to misleading conclusions that could undermine the next generations' well-being. This limitation is particularly critical when assessing emerging fuels with a potentially large environmental footprint at the Earth-system level due to their large production volumes and associated emissions.

The Planetary Boundary (PB) concept offers an appealing framework to quantify absolute sustainability precisely. The PBs represent a set of absolute global limits defined for the following Earth-system processes: climate change, ozone depletion, ocean acidification, nitrogen and phosphorus cycle, biodiversity and use of water and land resources. These biophysical limits should not be exceeded to operate within the Earth's carrying capacity.^28–30^ Nine PBs were put forward, which together define a safe operating space (SOS) for humanity. Going beyond the SOS may trigger irreversible events that could shift the planet's current equilibrium state. Research on the PBs has mostly focused on refining these limits and including additional Earth-system processes. In contrast, their application to the assessment of engineering systems (including fuels and energy systems) is still in its infancy, most likely due to the lack of appropriate assessment methods. In recent pioneering work, Ryberg and co-workers addressed this research gap by developing a method that contextualises the LCA results using the PB framework.^31^ This work has unfolded new avenues for evaluating a wide range of industrial systems through the lens of the PBs, including supply chains,^32^ chemicals,^33^ national economies,^34^ commercial products,^35^ and communities.^36^

The future transport sector will play a key role in sustainable development, yet the literature applying PBs in this context is very scarce. In this regard, the work by Bjørn *et al.*^37^ reviewing life-cycle based methods for quantifying absolute sustainability, highlights that most of the studied production and consumption activities exceed the assigned carrying capacity. However, the case studies identified in the review mainly cover the impacts of companies, nations and buildings and other areas of production, while none of them addresses the transport sector.

The goal of our study is to assess the performance of heavy-duty trucks powered by hydrogen to shed light on the global environmental footprint of meeting future transport needs with alternative fuels. In order to identify environmental criticalities for hydrogen as a global solution for heavy transport, LCA principles^11,12^ coupled with tailored impact assessment methods based on the PBs are applied to different hydrogen alternatives. To this end, we carry out a tailored LCA to quantify the life-cycle performance of heavy transport fuelled by hydrogen in relation to the absolute ecological limits of the Earth using the PB framework. Our LCA covers all the activities from well-to-wheel, from hydrogen production to its use as a fuel in trucks, focusing on quantifying their impact on the control variables of the PBs.^28–30^ Notably, we evaluate the life-cycle performance of heavy transport fuelled by hydrogen using the SOS defined by the PB framework. Hence, going well beyond standard LCAs, we analyse whether the hydrogen fuel would help humanity operate safely within the PBs. The LCA study covers seven hydrogen scenarios, *i.e.*, five electrolytic routes and two based on biomass gasification, and benchmarks them against the business-as-usual (BaU) scenario based on fossil diesel heavy-duty trucks.

These analyses are addressed in the Results and discussion section of the manuscript, which is preceded by the section describing the methodological steps. The last section of the article provides the conclusions of the work.

## Methodology

### Life cycle assessment

LCA quantifies the environmental impacts of products, processes, and services over their life cycle, covering a wide range of potential damages to identify environmental hotspots and improve overall environmental performance. Here we follow the ISO standards based on the four main LCA phases:

(i) “Goal and scope definition”, which defines the primary purpose of the analysis, the intended audience, time and geographical resolution, the system boundaries, and the primary function(s) of the system (*i.e.*, the functional unit [FU]). Given the present study's global scope, the FU corresponds to the global tonne km (t km) demand for road freight activities. According to projections starting from year 2015 made by the International Energy Agency,^6^ the demand is estimated to be *ca.* 33 trillion t km y^−1^ for 2020. However, note that recent statistics^41^ estimated that, due to the COVID emergency effects on the global economies, depending on the region, the travel volume of heavy transport suffered a reduction of up to 10%. Hence, our analysis provides more conservative (*i.e.*, pessimistic) results. Concerning the system boundaries, they cover from the feedstock and energy source production (to run the conversion process), up to the compression stage at 350 bar (*i.e.*, the delivery pressure for commercial hydrogen trucks equipped with a proton exchange membrane fuel cell [PEMFC]).^42^ The system boundaries also cover the manufacture of vehicles, construction of the production plant, and road use.

(ii) “Inventory analysis”, which quantifies the main inputs and outputs crossing the system boundaries (energy, raw materials, co-products, wastes, and emissions) and allocates them to the co-function(s) of the system. Specific details regarding the inventory analysis of the present study are reported in the section “Data collection and life-cycle models”.

(iii) “Impact assessment”, which quantifies the impact categories following specific impact assessment methods. This step consists of two mandatory sub-steps, namely, classification and characterisation. For the specific purpose of this study, the characterisation model relies on the factors calculated by Ryberg *et al.*^31^ that are consistent with the PB framework first defined by Rockström *et al.*^28^ and later updated by Steffen *et al.*^29^

(iv) “Interpretation”, which summarises the main conclusions and recommendations according to the study's goal and scope.

We stress that traditional LCA metrics are often used to rank and compare alternatives and benchmark new product designs *versus* a market reference. However, quantifying absolute environmental sustainability requires the explicit consideration of the planet's finite resources and carrying capacity.^43,44^ The PB concept defined by Rockström *et al.*^28^ (later updated by Steffen *et al.*^29^) provides absolute planetary limits and background levels for a set of nine critical Earth-system processes that can be used to quantify the absolute sustainability level of technologies. We shall use this framework together with Ryberg's method^31^ to quantify the absolute environmental sustainability level of hydrogen technologies.

### Connecting planetary boundaries to the life cycle inventory

Our work applies the characterisation factors proposed by Ryberg *et al.*^31^ for six PBs (leaving out atmospheric aerosol loading), and quantifies the biosphere integrity PB following Galán-Martín *et al.*^45^Table 1 summarises the seven Earth-system processes, their control variables (CVs, *i.e.*, the biophysical variables used to quantify the status and limits of each specific PB), their safe operating space level (SOS, *i.e.*, the difference between the boundary and the natural background, which indicates the budget for maximum anthropogenic perturbation without compromising the Earth system's stability) and current transgression levels (LTs), defined as the ratio between the current status of the CV (CS) and the respective SOS. It should be noted that the PB “atmospheric aerosol loading” is omitted because the original PB focuses only on the Indian subcontinent,^29^ while the scope of our analysis is global. Moreover, the PB “Chemical pollution and the release of novel entities” is excluded because it has not been defined yet, so both the control variables and specific characterization models are lacking. For seven of the nine CVs the current status already exceeds the SOS at the global level (*viz*., those with LT > 1). These current transgression levels highlight the need for policy action to comply with the planet's carrying capacity.

**Table:** Planetary boundary (PB), natural background level (NBL), safe operating space (SOS), current anthropogenic status (CS), and level of transgression (LT)

Earth-system process	Control variable (CV)	Abbr.	Unit	PB^29^	NBL^29,35^	SOS^29,35^	CS^29^	LT
Climate change	Atmospheric CO_2_ concentration	CO_2_	ppm CO_2_	350	278	72	1085^a^	15.1
Climate change	Energy imbalance at the top of the atmosphere	EI	W m^−2^	1	0	1	14.8^a^	14.8
Stratospheric ozone depletion	Stratospheric O_3_ concentration	O3D	DU	275	290	15	7	0.5
Ocean acidification	Carbonate ion concentration	OA	Ω_arag_	2.75	3.44	0.69	3.3^a^	4.8
Biogeochemical flows	P flow from freshwater to the ocean, global	P	Tg P y^−1^	11	1.1	9.9	20.9	2.1
Biogeochemical flows	Biological fixation of N, global	N	Tg N y^−1^	62	0	62	150	2.4
Land-system change	Area of forested land % of original, global	LSC	%	75	100	25	38	1.5
Freshwater use	Maximum consumptive blue water use, global	FWU	km^3^ y^−1^	4000	0	4000	2600	0.7
Change in biosphere integrity	Biodiversity intactness index	BII	% BII loss	10	0	10	26.8^b^	2.7

a Adjusted to yearly GHG emission data in 2015 from the EORA database^38^ and considering a time horizon until 2300 consistently with Ryberg *et al*.^31^

b Estimated from Sanchez-Ortiz *et al*.^39,40^

We quantify the performance of a set of scenarios, each entailing a different fuel production process, in seven Earth-system processes linked to nine CVs. A common approach to quantify absolute sustainability consists of applying downscaling principles to assign a share of the SOS to the specific system under study.^37^ If the system exceeds the allocated ecological budget, it is deemed unsustainable (and sustainable otherwise). However, these sharing principles are controversial, and there is not yet a universal agreement on which one should be applied in practice. To avoid downscaling methods in the interpretation phase, we quantify an aggregated PB performance metric that simulates the global transgression level that would result from replacing the BaU scenario with an alternative one, as we did in previous work of some of us.^45^ To this end, we subtract from the current total anthropogenic impact the contribution of the BaU (*i.e.*, of the current transport sector) and add the impact of the alternative scenario. That is:

1 EB^GLO^_*b*,*s*_ = EB^CUR^_*b*_ − EB_*b*,BaU_ + EB_*b*,*s*_ ∀*b* ∈ *B*, *s* ∈ *S*

where *S* is the set of alternative scenarios (*i.e.*, based on the seven hydrogen production pathways); *B* is the set of CVs (Table 1); EB^GLO^_*b*,*s*_ is the global impact in CV *b* under the scenario *s*; EB_*b*,BaU_ is the impact of the BaU in CV *b*; and EB_*b*,*s*_ is the impact of the scenario *s* in CV *b*. We then recalculate the global level of transgression (LT) in each CV for every scenario as follows:2
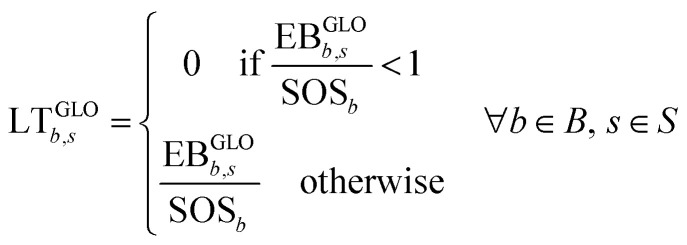
where LT^GLO^_*b*,*s*_ is zero if the CV is not exceeded or is given by the quotient between the impact and the SOS otherwise. We finally define the aggregated transgression level for each scenario as follows:3
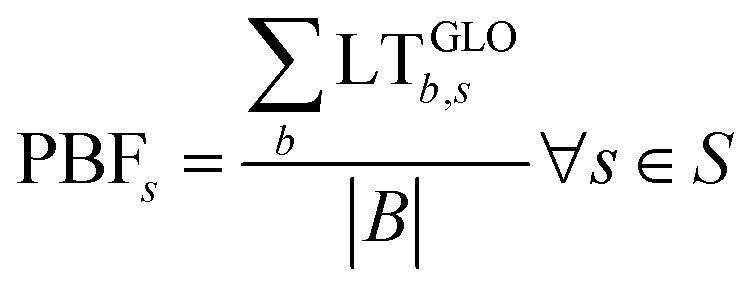
where PBF_s_ is the PB footprint of scenario *s*, and the cardinality of set *B* (|B|) provides the number of CVs (*i.e.*, nine in this work). The impact of a scenario in each Earth-system process and the aggregated metric are finally used to interpret the PB results.

Furthermore, given the increasing role of climate-change-related aspects in the current policy-making context, we complement the analysis of the aggregated indicator PBF using two additional sub-indicators aggregating, respectively, the GHG-driven PBs (whose CVs are CO_2_, EI, and OA) and the other PBs, as follows:4
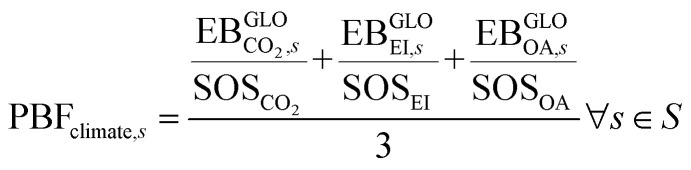
5
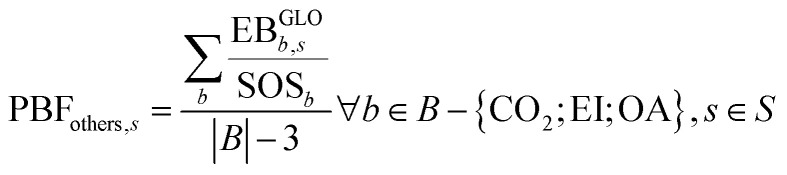
where, for each scenario *s*, eqn (4) provides the average transgression of the GHG-driven PBs (*i.e.*, CVs of CO_2_, EI and OA), considering that these CVs are equally important, and eqn (5) is the average transgression of the scenario in the remaining PBs. It should be observed that ocean acidification is caused by the accumulation of carbonic acid in oceans, so it is closely connected to the increase in atmospheric CO_2_ concentration, which in turn is linked to carbon emissions.^31^ For this reason, in the characterization model developed by Ryberg *et al.,*^31^ the same elementary flows affect the CVs for CO_2_ concentration and ocean acidification.

### Technologies and scenarios

**Fig. 1:**
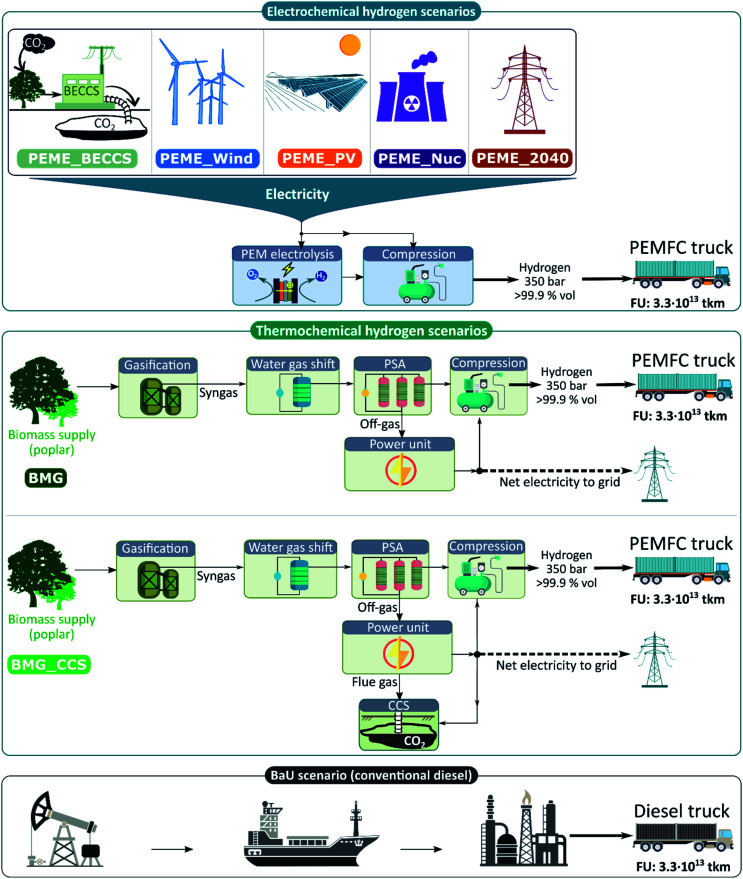
Scope and technologies of the scenarios under analysis.

Table 2 summarises the main features of the seven hydrogen production pathways, *i.e.*, feedstock, energy sources, conversion technology and co-products, and Carbon Capture and Storage (CCS) systems. Two leading conversion technologies are involved, namely water electrolysis and biomass gasification. Fig. 1 shows the technologies' scope, distinguishing between electrochemical (based on electrolysis) and thermochemical (biomass gasification) pathways. The seven hydrogen alternatives are benchmarked *versus* the BaU scenario based on conventional diesel for heavy transport. The hydrogen production pathways involve the use of non-fossil energy or feedstock with a minimum technology readiness level equal to 5, *i.e.*, validated technologies relevant at an industrial level.^46^

**Table:** Main features of the H_2_-fuel alternatives under analysis

Case code	Technology	Feedstock	Energy source	Co-product	CCS
BMG	Gasification	Poplar	Biomass	Electricity^a^	—
BMG_CCS	Gasification	Poplar	Biomass	—	MEA
PEME_BECCS	Electrolysis	Water	Biomass-based power (poplar)	—	MEA
PEME_Wind	Electrolysis	Water	Wind power	—	—
PEME_PV	Electrolysis	Water	PV power	—	—
PEME_Nuc	Electrolysis	Water	Nuclear power	—	—
PEME_2040	Electrolysis	Water	Global 2040 mix^b^	—	—

a System expansion approach is followed to deal with multifunctionality.

b Based on the IEA World Energy Outlook, 2019, Sustainable Development scenario.^47^

In biomass gasification (endothermic process), biomass supplies the required energy, acting as a hydrogen carrier. The gasification process follows four main steps (dehumidification, pyrolysis, gasification, and combustion), which occur in the reactor generating hydrogen-rich syngas. The syngas composition (mainly composed of H_2_ and CO) is a critical variable that depends on several factors (*e.g.*, biomass composition, gasification technology, and gasifying agent).^48^ The syngas undergoes the water gas shift reaction followed by pressure swing absorption (PSA) to reach a hydrogen purity above 99.9% (vol).^49^

The biomass feedstock considered is a short-rotation poplar coppice, a widespread second-generation bioenergy crop.^50–52^ We model biomass gasification with and without the CCS system (BMG and BMG_CCS, respectively), where the CCS sub-system is based on post-combustion capture with a monoethanolamine solvent (MEA).

Water electrolysis produces high-purity hydrogen by dissociating the H_2_O molecule into ions under the effect of a direct current supplied to the electrodes. Electrolysers differ in the type of electrolyte used in the cell. In proton exchange membrane electrolysis (PEME), the electrolyte is a polymeric membrane (perfluoro-sulphonic acid) that distributes the ions between the anodic and cathodic compartments. The power sources often considered in LCA studies of hydrogen energy systems include wind electricity, photovoltaic, nuclear, and grid mix electricity. In contrast, studies dealing with biomass-based electricity are scarce.^25^ Nevertheless, given the growing interest in bioenergy with carbon capture and storage (BECCS) as a crucial CO_2_-negative technology to mitigate global warming,^53–55^ this work covers PEME powered by BECCS (PEME_BECCS). Here electricity is supplied by a biomass-based combined heat and power plant, adopting for CCS the same assumptions as in BMG_CCS. The captured CO_2_ is transported over 200 km and finally injected into a depleted gas field well for its geological storage.

At present, several technical barriers hinder the large-scale deployment of hydrogen (*e.g.*, high production costs and lack of infrastructure).^5,56^ However, significant efforts aim to overcome these barriers and enhance the hydrogen's strategic role in the mid-term economy.^5,9,57–59^ Given this context, we focus on the technological trends expected in the mid-run (in 2040 for all the scenarios) rather than the current state-of-the-art technologies.

To this end, future prospects for key technical features that could potentially have a significant influence on the results of the PB study (*e.g.*, electricity mix, vehicle consumption, technical efficiency, and lifetime of key units) are considered for both the foreground and the background system (Table 3). However, for simplicity, we modified only a set of background processes restricted to the most energy-consuming activities. In particular, the projected electricity mix of 2040 (defined based on the “Sustainable Development scenario” from the IEA^47^) was considered for the background processes contributing the most to the total electricity demand over the life cycle. These processes include base metal (steel, aluminium, titanium, and copper) and fuel (oil, natural gas, coal, and diesel) production routes used in some sub-components (namely, in bipolar plates, catalysts, gas diffusion layers, polymeric membranes, and the assembly of PEME and PEMFC stacks, as well as in carbon fibre production for hydrogen tanks). Notably, the electricity demand of such activities, when summed to the direct electricity consumption of the foreground system, was found to be responsible for 74–98% of the total cumulated electricity consumption. In turn, the impacts embodied in the future electricity mix were calculated based on its composition and the efficiencies of its technologies (Table 3).

Evolution for technical parameters (the baseline refers to 2040)

**Table:** 

Parameter	Current value	2040 value
Efficiency, gasification (biomass)^70^	35%	47%
Efficiency, CHP (syngas)^70^	53%	62%
Efficiency, combined cycle (NG)^70^	58%	62%
Efficiency, coal power plant^70^	45%	48%
Efficiency, furnace steam turbine^70^	34%	38%
Efficiency, solar PV system^70^	17%	25%
Capacity factor, CSP power^70^	38%	41%
Capacity factor, onshore wind turbine^70^	30%	40%
Capacity factor, offshore wind turbine^70^	40%	48%
Efficiency, geothermal (ORC)^70^	13.8%	14.7%
Efficiency, PEM electrolyser^9^	0.61	0.70
Lifespan, PEM electrolyser^9^	40 000 h	60 000 h
Diesel consumption (diesel truck)^a^	36.7 g t km^−1^	33.6 g t km^−1^
H_2_ consumption (PEMFC truck)^b^	2.4 g t km^−1^	2.1 g t km^−1^

a Current value based on the Ecoinvent model for “Lorry 16–32 metric ton, EURO6 {RER}”; 2040 value calculated by considering an improvement of 10% in fuel economy (ratio “ultimate” to “interim” value under the conservative assumption for a Class 8 tractor-trailer in Marcinkoski *et al*., 2019)^71^

b Current value based on commercial H_2_ truck information (Hyundai XCIENT fuel cell heavy-duty truck);^65^ 2040 value calculated by considering improvement of 12% in fuel economy (ratio “ultimate” to “interim” value under the conservative assumption for a Class 8 tractor-trailer in Marcinkoski *et al*., 2019)^71^

**Table:** 

Electricity mix composition^47^		
Coal	38.11%	3.71%
Natural gas	23.03%	12.09%
Oil	3.04%	0.51%
Nuclear	10.23%	11.39%
Hydro	15.82%	17.99%
Bioenergy	2.39%	5.69%
Wind	4.76%	21.49%
PV	2.23%	18.69%
Geothermal	0.34%	1.43%
Concentrated solar	0.04%	2.08%
Coal + CCS	0.00%	2.57%
Natural gas + CCS	0.00%	2.37%

### Data collection and life-cycle models

Data for the poplar biomass gasification process were retrieved from Susmozas *et al.*^60^ The upstream process of the poplar cultivation activity is based on the model defined in Peters *et al.*,^61^ which accounts for the CO_2_ emissions due to land transformation. The CCS system in post-combustion is modelled according to Volkart *et al.*,^45^ and considering the co-benefits of CCS regarding reductions in CO, SO_2_ and NO_x_ emissions, as reported in Koornneef *et al.*^46^ The CO_2_ transportation and storage was modelled following Wildbolz *et al.*^62^ considering transportation *via* a pipeline over 200 km and geological storage in a depleted gas field well. The LCA model of PEM electrolysis is based on Bareiß *at al.*^63^ for both operation and infrastructure. Regarding trucks, the BaU scenario considers the system “Lorry 16–32 metric ton, EURO6” of the Ecoinvent v3.5 database.^64^ The infrastructure and operation of the PEMFC truck were adjusted considering the datasheet of the Hyundai XCIENT Fuel Cell truck.^42^ The models for the specific sub-activities of PEMFC transport technologies were modelled according to the scientific literature and proportionally scaled to meet the PEMFC truck specifications.^65^ Notably, the inventory data for the PEMFC stack, hydrogen tank, electric motor and battery pack are based on Simons and Bauer,^21^ Evangelisti *et al.*,^66^ Notter *et al.*,^67^ and Ellingsen *et al.*^68^

The life-cycle models for all the scenarios were implemented in the SimaPro 9 software^69^ using Ecoinvent 3.5 (ref. 64) as the data source for the background processes. In order to characterise both the foreground and the background system, the specific LCIA method was implemented in the SimaPro software considering all the LCI entries over the life cycle and using the characterization factors provided in Ryberg *et al.*^31^

### Baseline and sensitivity to key parameters

Table 4 shows the list of parameters and associated ranges considered in the sensitivity analysis, which provide insight into their accuracy within the uncertain parameter space. The technological parameters of Table 3 used in the default calculations (baseline) refer to 2040 values. The soil organic carbon stock (SOC) in biomass cultivation is varied within the range −15 to +5 Mg of soil C sequestered per hectare of land to model the uncertainty linked to the CO_2_ emissions due to land-use change (more pronounced in the scenarios involving biomass cultivation in the foreground system, *viz*. BMG, BMG_CCS, and PEME_BECCS). This range is estimated based on how much the absolute SOC changes due to different land type transformations. *i.e.*, from forest, farmland, and grassland to forest, as quantified by Deng *et al.*^72^ The baseline of poplar cultivation considers a variation of around −10 Mg C per hectare, derived from Peters *et al.*^61^ assuming that the poplar plantation is on degraded land. In the biomass-based case studies, we investigated the effect of uncertainties in the SOC, plantation lifetime and biomass yield. Moreover, in the cases involving electrolysis, we considered uncertainties in the electrolyser lifetime and efficiency, where the baseline scenario assumes conservative values for both the electrolyser efficiency and lifetime. The values for the best and worst scenarios for PEM electrolysis are based on a recent expert elicitation study concluding that these technical parameters could vary significantly.^73^ Concerning the vehicle performance, the baseline case considers conservative values for fuel consumption; however, more optimistic values reported in Marcinkoski *et al.*^71^ are here used to model the best scenario.

**Table:** Values of the parameters in the baseline, best and worst scenarios

Parameter	Unit	Baseline	Best case	Worst case
SOC	Mg C ha^−1^	−10	−15	5
Crop yield	t biomass ha^−1^	13.5	16	11
Plantation lifetime	years	15	25	10
PEME lifetime	hours	60 000	125000	40 000
PEME efficiency	% (LHV)	70	77	60
Diesel consumption	g diesel t km^−1^	33.6	23.5	33.6
H_2_ consumption	g H_2_ t km^−1^	2.1	1.5	2.1

## Results and discussion

The following section summarises the impact on the PBs of the seven hydrogen-to-truck routes. For benchmarking purposes, we also show the results of the BaU, *i.e.*, a conventional diesel truck.

### Relative contribution to the safe operating space

**Fig. 2:**
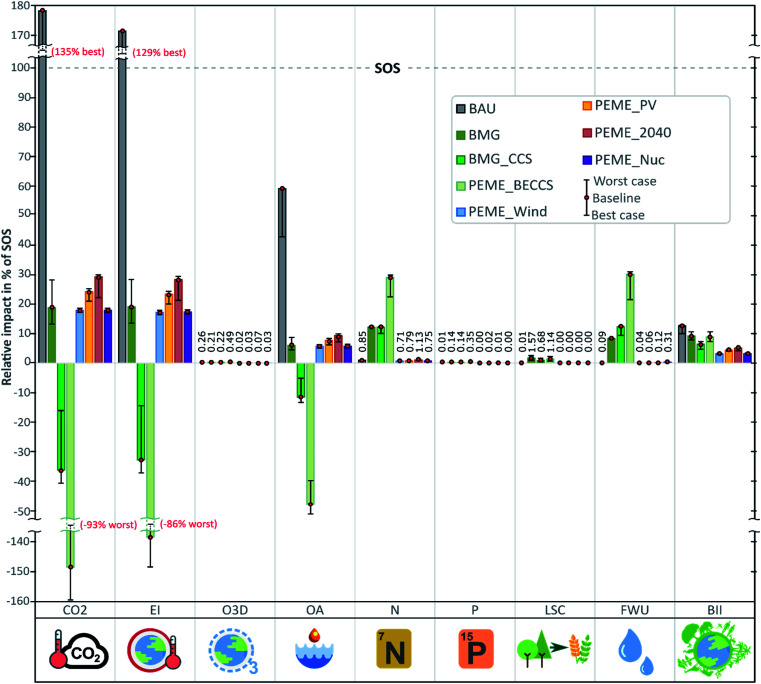
Share of the SOS for the diesel-based (BaU) scenario and H_2_-based scenarios for heavy transportation. The results refer to 2040. Values over 100% indicate a performance transgressing the planetary boundary. Deviation bar denote the best and worst cases (Table 4). (CO_2_: atmospheric CO_2_ concentration; EI: energy imbalance at the top of the atmosphere; O3D: stratospheric ozone depletion; OA: ocean acidification; P: biogeochemical phosphorus flow, global; N: biogeochemical nitrogen flow, global; LSC: land-system change, global; FWU: freshwater use, global; BII: biosphere integrity). The separate contributions of the land use and climate change stressors to BII are reported in Fig. A1 in the appendix.†

We first analyse the impacts of all the scenarios (*viz.*, the impacts embodied in the global t km demand from the heavy transport sector satisfied by each fuel alternative). Fig. 2 shows the percentage of the SOS (*i.e.*, total budget available for the global economy) occupied by the transport sector in the PBs addressed (relative contribution given by the ratio EB_*b*,*s*_/SOS_*b*_ for control variable *b* and scenario *s*). The figure also reports the interval defined by the best and worst cases. Values above 100% indicate that the technology transgresses the global PB and, therefore, is unsustainable in that Earth-system process.

The results show that the BaU heavy transportation sector alone already transgresses the global SOS in CO_2_ concentration and energy imbalance at the top-of-the-atmosphere (both strongly linked to greenhouse gas emissions) by a factor of around 1.7. Furthermore, the BaU scenario occupies a large share of the SOS in other PBs, yet it does not fully transgress them, *i.e.*, 58% in ocean acidification and 13% in biosphere integrity, both also associated with GHG emissions. The contribution of the BaU to the other PBs is always below 1%.

None of the hydrogen-based alternatives transgresses the SOS of the PBs. Therefore, hydrogen can be considered an environmentally sustainable alternative to conventional diesel as a fuel for heavy transportation. However, burden-shifting does occur in some alternatives, *e.g.*, nitrogen flow and freshwater use worsen substantially in the biomass-based alternatives (12% in BMG, 12% in BMG_CCS and 29% in PEME_BECCS of the SOS defined for N-flow; 8% in BMG, 12% in BMG_CCS and 30% in PEME_BECCS of the SOS available for freshwater use). This worsening is mostly due to the fertilisers and irrigation needed for biomass growth.^61^ In this regard, it has to be highlighted that the burden shifting in biomass-based pathways could be alleviated if forest residues or residues from less intensive plantations were used instead of dedicated poplar plantations. However, the global availability of residues from the forestry production and trade (<500 Mt per year according to FAO statistics)^74^ would not be sufficient to cover the specific requirements of the gasification system (*i.e.*, around 2500 Mt per year).

Technologies involving CCS (*i.e.*, PEME_BECCS and BMG_CCS) show net benefits for the climate change PB, with CVs of atmospheric CO_2_ concentration and energy imbalance at the top of the atmosphere, and the ocean acidification PB. Their negative carbon balance implies that they could be implemented as core strategies for decarbonising heavy and long-distance transport. These CCS-based scenarios, however, lead to burden shifting in the biogeochemical nitrogen flow (mainly associated with the use of fertilisers for poplar cultivation) and biosphere integrity (associated with land use), both currently transgressed at the global level (Table 1). With regard to biosphere integrity, note that our methodology considers two main stressors, *i.e.* GHG emissions and land use.^45^ Both PEME_BECCS and BMG_CCS show negative CO_2_ emissions (leading to savings in biodiversity), yet their high land-use requirements result in poor biodiversity performance (Fig. A1 in the appendix†).

Notably, the impacts in the land-system change (LSC) PB are <1% for the three biomass-based options (PEME_BECCS, BMG and BMG_CCS). Note that the control variable used in the PB framework^28,29^ refers to the amount of forest cover remaining. Accordingly, the characterization model developed by Ryberg *et al.*^31^ considers forest transformation solely. Hence, no LSC impacts are accounted for during the poplar cultivation stage in the biomass-based options due to the transformation assumed (from degraded land). Nevertheless, in order to check the representativeness at the global scale of these scenarios (based on a country-specific geographical context),^61^ we performed an additional analysis on the marginal land globally available. In this regard, around 44 million hectares of marginal land are required to cultivate the amount of biomass needed to obtain the hydrogen fuel from biomass gasification. Such an area is estimated to range within 3–14% of the marginal land globally available calculated by Cai *et al.*;^75^ these results, therefore, confirm the technical feasibility of the biomass-based hydrogen scenarios at the global scale.

In terms of GHG-related PBs, we find that PEME_BECCS outperforms BMG_CCS; however, the former shows higher impacts on the other PBs. This finding can be explained by the biomass-to-H_2_ efficiency, lower in PEME_BECCS, *i.e.*, more biomass needed per unit of hydrogen produced. On the downside, this low efficiency in converting biomass into hydrogen exacerbates other PBs and, potentially, other sustainability dimensions, *e.g.*, economic and social impacts.^76^ Notably, higher amounts of woody biomass (poplar) result in higher CO_2_ uptake benefits in climate change PBs, but require more resources (fertilisers, land, and pesticides) with adverse effects on other PBs. In this regard, decision and policy makers should not overlook this trade-off when deploying 2nd generation biomass-based fuels.

Focusing on the scenarios involving electrolysis, they generally show a better performance when compared with the BaU scenario in all the PBs, excluding freshwater use (mainly due to the water requirements in the electrolytic process, *i.e.*, 9 kg water per kg of hydrogen^63^). However, currently, with a non-transgressed PB, this burden shifting should not be that critical from an absolute sustainability standpoint. It should be noted that the nuclear-based scenario shows a profile similar to that of the wind path. However, the critical aspects of nuclear energy (*viz*. nuclear waste management, nuclear risk, externalities, and public acceptance),^77^ which may overturn the conclusions on the suitability of nuclear-based hydrogen, are out of the scope of the present analysis.

Among the hydrogen alternatives (*i.e.*, excluding the BaU), the scenario based on electrolytic hydrogen from the grid electricity (PEME_2040) leads to the highest impacts in all of the PBs, except for those PBs whose impacts are driven mostly by biomass cultivation (*i.e.*, the N flow and biosphere integrity PBs). Although an optimistic share of renewable electricity has been adopted (67.4% of renewable, 11.4% of nuclear, 16.3% of fossil, and 4.9% of fossil with CCS),^47^ the hydrogen from the grid tends to be environmentally inferior in the climate PBs with respect to the other hydrogen scenarios but still beneficial when compared to the BaU. Nevertheless, when comparing the 2040 scenarios (baseline) with the current state-of-the-art (Fig. A2 in the appendix†), though an improvement of the environmental performance is generally expected for all the hydrogen technologies, the benefits from the technological advances are more evident for the PEME_2040 case study in seven out of the nine control variables. In 2040, electrolysis impacts in the biogeochemical flow PB (N- and P-flow) are estimated to be higher than those considering current technologies due to the increased amount of biomass-based electricity in the 2040 mix (Table 3). Apart from the biogeochemical PB, the savings in relative impacts of the grid-based electrolysis in 2040 with respect to the current grid-based electrolysis are estimated to be in the range of 75% (in GHG-related PBs) to 40% (in the land system change PB). Notably, remarkable improvements (ranging from 20 to 40% of impact savings) are estimated for the PV-based pathways in all of the PBs. In contrast, the BMG_CCS alternative would slightly worsen in the GHG-related PBs due to the improvement in the biomass gasification efficiency in 2040, which would reduce the CO_2_ uptake per FU.

Among the scenarios based on electrolytic hydrogen, wind power electrolysis is identified as the most promising renewable alternative. Disregarding the carbon-negative technologies, which attain the best performance in the GHG-related PBs at the expense of a severe burden shifting in BII and N flow CVs, the wind-based hydrogen fuel performs best in all the PBs with respect to both the BaU scenario and the alternative ones. This aspect, however, is addressed in more detail in the next section that provides the PBF indicators, based on which the scenarios can be ranked.

Finally, moving from the baseline to the best and worst-case scenarios by varying the parameters according to Table 4 can affect the technological ranking in some PBs. For instance, as can be observed in Fig. 2, an uncertainty range exists within which PEME_2040 could outperform PEME_PV in the CVs linked (fully or in part) to GHG emissions (namely, CO2, EI, OA, and BII). Similarly, the performance of the biomass without CCS (BMG) varies remarkably due to the CO_2_ uptake in the poplar cultivation. Under the best scenario, this alternative would outperform the renewable electrolysis-based ones in the climate change PB. However, in both the best and worst cases, the same burden-shifting and breakdown patterns are observed, while the insights concerning the suitability of hydrogen scenarios *versus* the BaU remain the same.

Overall, the PB analysis showed the potential environmental benefits under absolute criteria of the implementation of a hydrogen-based economy in heavy transport. We identified wind-based hydrogen fuel as a generally favourable alternative pathway. Besides, although the carbon-negative technologies show the highest SOS potential savings, they cannot be unequivocally selected as the best solution under the complete set of PBs addressed due to their large collateral damage in other PBs already transgressed.

### Main impact contributions

**Fig. 3:**
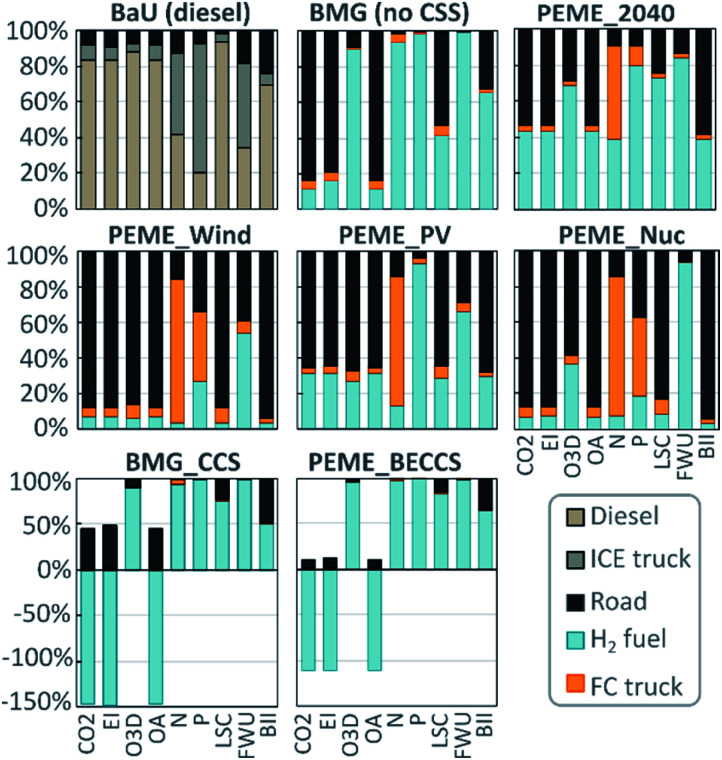
Impact breakdown of the case studies analysed. In these figures, 100% represents the total impact of the scenario in the specific Earth-system process (abbreviations in Table 1). Diesel and hydrogen fuels include the direct emissions of the use phase.

Fig. 3 shows the relative impact breakdown in the nine CVs for the scenarios analysed. Though in absolute terms, capital goods and infrastructure have similar contributions in all the scenarios, in relative terms road use is found to be among the main hotspots in all the PBs for the hydrogen fuel scenarios.

In contrast, road use plays a minor role in the BaU scenario, where diesel use mostly dictates the impact. The high relative contribution of capital goods and infrastructure in the hydrogen scenarios is due to the land requirements and the use of fossil resources (bitumen and diesel) in road construction, with adverse effects on ecosystems and the climate change-related PBs.

Fig. 3 also shows that the burdens embodied in road use – as a relative contribution – are especially relevant in the electrochemical pathways. In particular, in the nuclear- and wind-based scenarios, all the impacts (excluding freshwater use) are mostly linked to truck manufacture and road construction manufacture rather than to hydrogen production, as in the other scenarios.

It should be noted that the transport infrastructure affects all the alternative scenarios by the same absolute value. Besides, we note that the main driver to the relatively higher impacts in the PV-based scenario is associated with the PV technology's relatively low efficiency. This low efficiency, in turn, leads to larger consumption rates of resources, mainly land use (driving the BII indicator) and raw materials required for manufacturing the PV modules.

In the production of electrolytic hydrogen, the mining of metals used for electronic components and the manufacturing of titanium in bipolar plates (both used in the fuel cell truck) are responsible for a large share of the impact on the biochemical N and P flow PBs (Fig. 3). However, these impacts are negligible in absolute terms (Fig. 2).

### Planetary boundary footprint

**Fig. 4:**
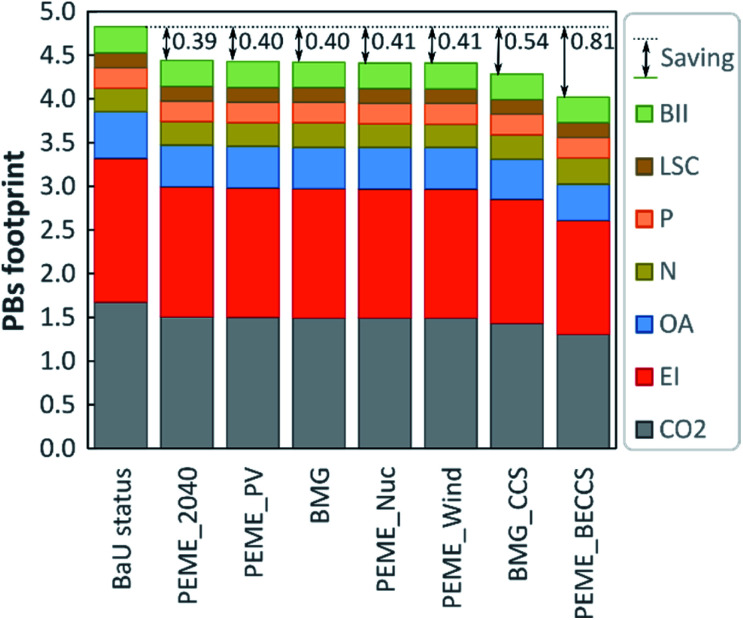
PBs footprint indicator for the hydrogen scenario (the breakdown shows the contributions of the transgression levels in each PB towards the average transgression level).

**Fig. 5:**
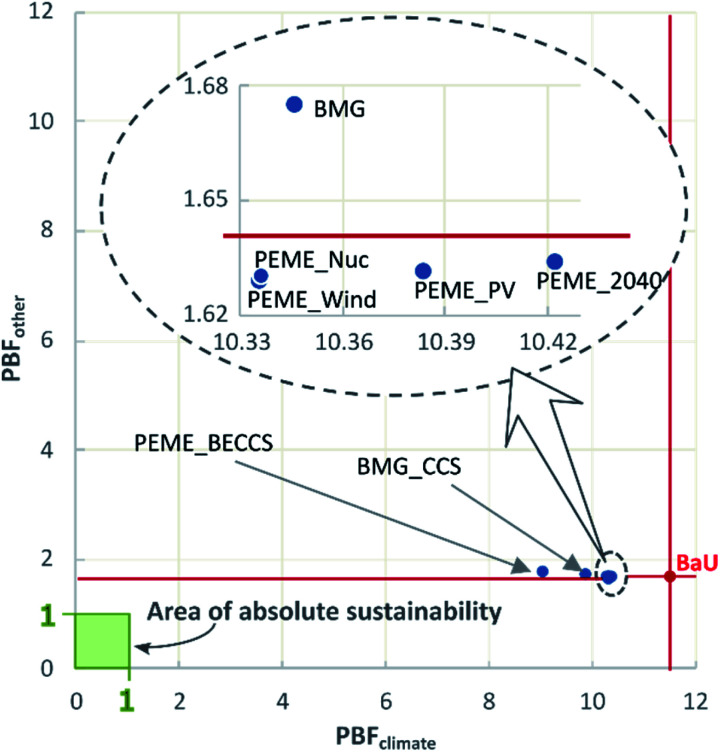
Average transgression levels in the climate-related and other PBs for each scenario.

We next compute the aggregated transgression metric defined earlier, as shown in Fig. 4. The figure is arranged in the descending order (left-to-right) of PBF values (*i.e.*, from the least favourable to the most favourable scenario). The PB footprint, as previously defined in eqn (3), represents the average global transgression aggregated through the sum of the transgressed CVs over the total number of CVs (transgressed and not). Also, given the magnitude of the transgression levels of the climate-related PBs (Fig. 2), ranging from high positive (in BaU) to high negative values (in BMG_CCS and PEME_BECCS), we propose to perform a separate analysis of the average transgression associated with the GHG-related PBs and the average transgression of the other PBs (Fig. 5).

When considering the sum of the transgressed CVs (Table 1) over the nine CVs (eqn (3)), the current PB footprint is around 4.8 times the SOS, meaning that on average, in the BaU, we transgress almost five times the PBs. The GHG-related PBs dominate the total transgression level. Hence, scenarios attaining the highest GHG-emission reductions perform best in this metric (*i.e.*, PEME_BECCS and BMG_CCS).

Indeed, the best performance in terms of average global transgression (PBF) is 4.0, followed by 4.3, and then 4.4 for PEM_BECCS, BMG_CC and PEME_Wind, respectively. However, the best performing scenarios exacerbate substantially impacts on other PBs (*e.g.*, N biogeochemical flow), which became clear when analysing the share of the SOS occupied by each alternative. In contrast, wind- and nuclear-based hydrogen led to lower global savings (*i.e.*, 0.4) but entail negligible burden shifting.

We next analyse the disaggregated average transgression levels, *i.e.*, of the GHG-related PBs (PBF_climate_) and of the other PBs (PBF_other_). In Fig. 5, PBF_climate_ and PBF_others_, calculated using eqn (4) and (5), represent the global transgression in a specific set of PBs, considering the substitution of the BaU with the respective alternative scenario. The green area denotes the zone of absolute sustainability within which there is no transgression of the aggregated PB indeces.

To interpret the figure, we apply the concept of Pareto optimality, *i.e.*, a solution is Pareto optimal if there is no other point that improves it simultaneously in all the performance metrics. As seen, the BaU is clearly suboptimal from an environmental standpoint, as there are several alternative scenarios (denoted by blue points) outperforming it in the two metrics simultaneously. Notably, all the electrolytic scenarios show a lower impact on the GHG-related and non GHG-related PBs, relative to the BaU. The Pareto frontier of this analysis would be given by PEME_BECCS, BMG_CCS, and PEME_Wind, with the other scenarios being suboptimal from a PB standpoint.

Nevertheless, it can be observed from Fig. 5 that none of the scenarios would allow operating within the area of absolute sustainability. In particular, the distance from the sustainability zone is generally larger in the climate-related axis for all the scenarios, stressing the need for prioritising climate-change-mitigating actions over other areas of impact. In this sense, only electrochemical technologies reduce the impact under both global indicators. However, in the options involving CCS, a relatively low increase in PBF_other_ could be accepted given their larger PBF_climate_ savings. In other words, despite the fact that biomass-based paths require technological improvements to fully outperform the BaU, in the mid-run, an optimal portfolio of technologies might be required to foster the deployment of hydrogen as a fuel in the transport sector.

## Conclusions

Our study quantified the environmental performance of hydrogen as an alternative fuel for heavy transportation in relation to the absolute ecological limits of the Earth (PBs). The application of LCA principles coupled with tailored impact assessment methods based on the PBs allowed the identification of environmental criticalities for the hydrogen roll-out as a global solution for future heavy transport.

The comparison under the PB metrics of the hydrogen fuel scenarios *versus* the BaU scenario (diesel trucks) showed a generally favourable hydrogen performance. In particular, through the lens of the PB framework, hydrogen produced by electrolysis was identified as the most promising technology at the expense of a marginal burden shifting in PBs currently not transgressed (*i.e.*, freshwater use).

When focusing on the PBs related to GHG (climate change and ocean acidification), the savings in CO_2_ emissions are found to drive the overall performance. In this regard, the case studies involving biomass and CO_2_ capture show the best overall performance. However, burden shifting in PBs currently transgressed (biogeochemical flow of N and biosphere integrity) is found in these options, raising potential concerns for their implementation. The uncertainty associated with relevant technical parameters is found to potentially affect the technology ranking under the climate-related PBs. However, the main overall conclusions would remain valid.

Overall, given the superior performance of the seven hydrogen-based scenarios relative to the BaU scenario, feeding heavy trucks with green hydrogen produced through a mix of mature technologies would significantly reduce the anthropogenic pressure on the environment, helping the transport sector to operate without transgressing the Earth's ecological limits. The scope of our study could be enlarged to embrace other economic activities beyond the transport sector and simulate a collective action aimed at operating within a zone of absolute sustainability for the Earth.

Our work can provide the basis for other PB studies aimed at evaluating the effects of emerging technologies on a global scale. Furthermore, further efforts are required to evaluate hydrogen's suitability as a fully sustainable fuel for heavy transportation. Along these lines, our analysis paves the way for future work that could integrate PBs and socio-economic metrics, including human health impacts and indicators derived from a social LCA, to design the optimal production mix for hydrogen in the transport sector. This would also require combining such metrics with optimization models to find optimal pathways to operate sustainably within the transport sector and beyond.

## Conflicts of interest

There are no conflicts to declare.

## Supplementary Material

SE-005-D1SE00790D-s001

SE-005-D1SE00790D-s002

SE-005-D1SE00790D-s003
