# Supplementary material for: The role of hydrogen in heavy transport to operate within planetary boundaries
Source: Sustain Energy Fuels. 2021 Jul 30;5(18):4637–49. doi: 10.1039/d1se00790d (PMC8439148; doi:10.1039/d1se00790d)
Supplement: SE-005-D1SE00790D-s001 [file SE-005-D1SE00790D-s001.pdf]

Electronic Supplementary Material (ESI) for Sustainable Energy & Fuels.
This journal is © The Royal Society of Chemistry 2021

## Appendix

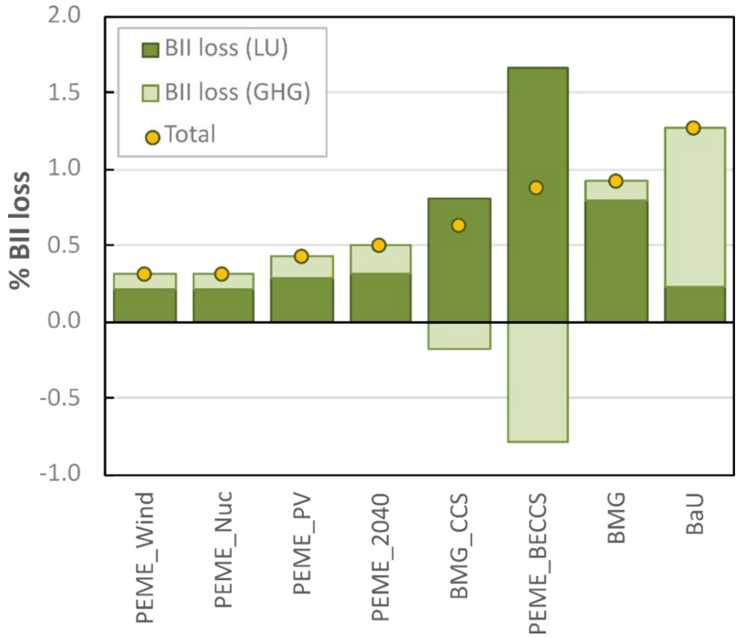


**Figure A1** Contribution of the environmental stressors to the PB of biosphere integrity. The bars are arranged in ascending order of total impact. (**BII**: biodiversity intactness index; **LU**: land use stressor; **GHG**: greenhouse gas emissions stressor).

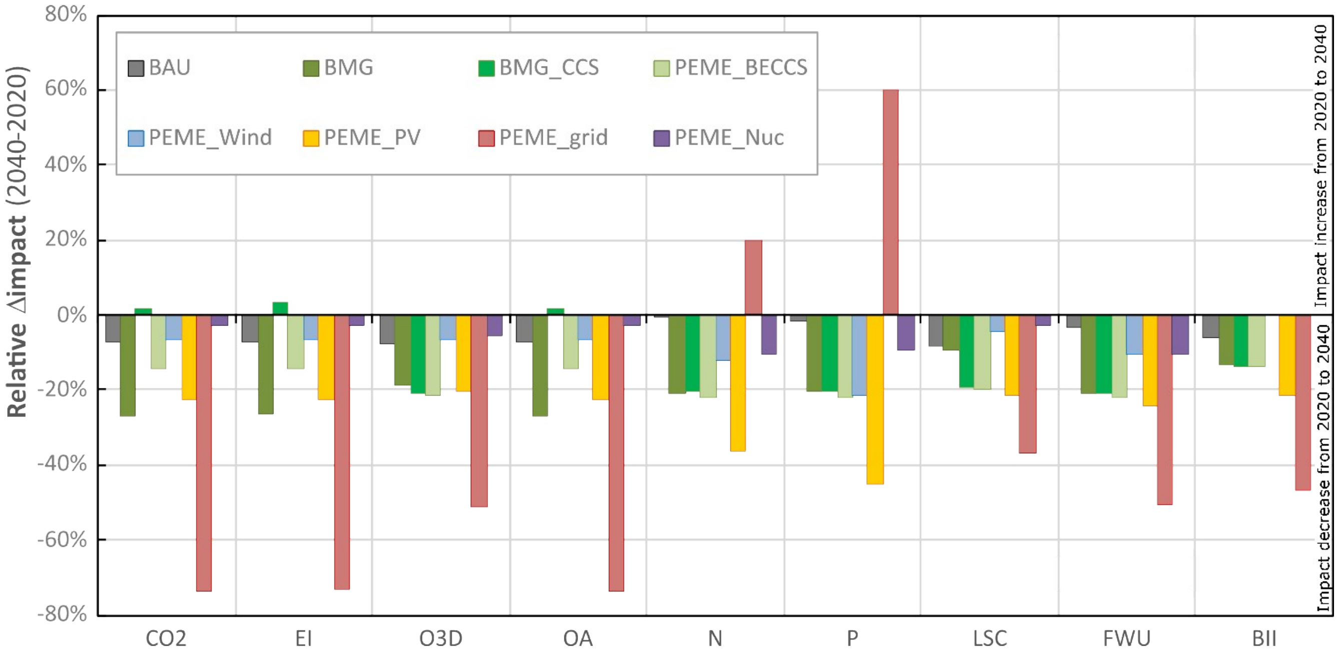


**Figure A2** Relative impact variation between 2040 and 2020 scenarios. Negative values denote better performance for the 2040 scenario under the specific control variables of the PBs. (**CO2**: Atmospheric $CO_2$ concentration; **EI**: Energy imbalance at the top of atmosphere; **O3D**: Stratospheric ozone depletion; **OA**: Ocean acidification; **P**: Biogeochemical phosphorus flow, global; **N**: Biogeochemical nitrogen flow, global; **LSC**: Land-system change, global; **FWU**: Freshwater use, global; **BII**: Biosphere integrity).
