# Supplementary figures and images for: The role of hydrogen in heavy transport to operate within planetary boundaries
Source: Sustain Energy Fuels. 2021 Jul 30;5(18):4637–49. doi: 10.1039/d1se00790d (PMC8439148; doi:10.1039/d1se00790d)

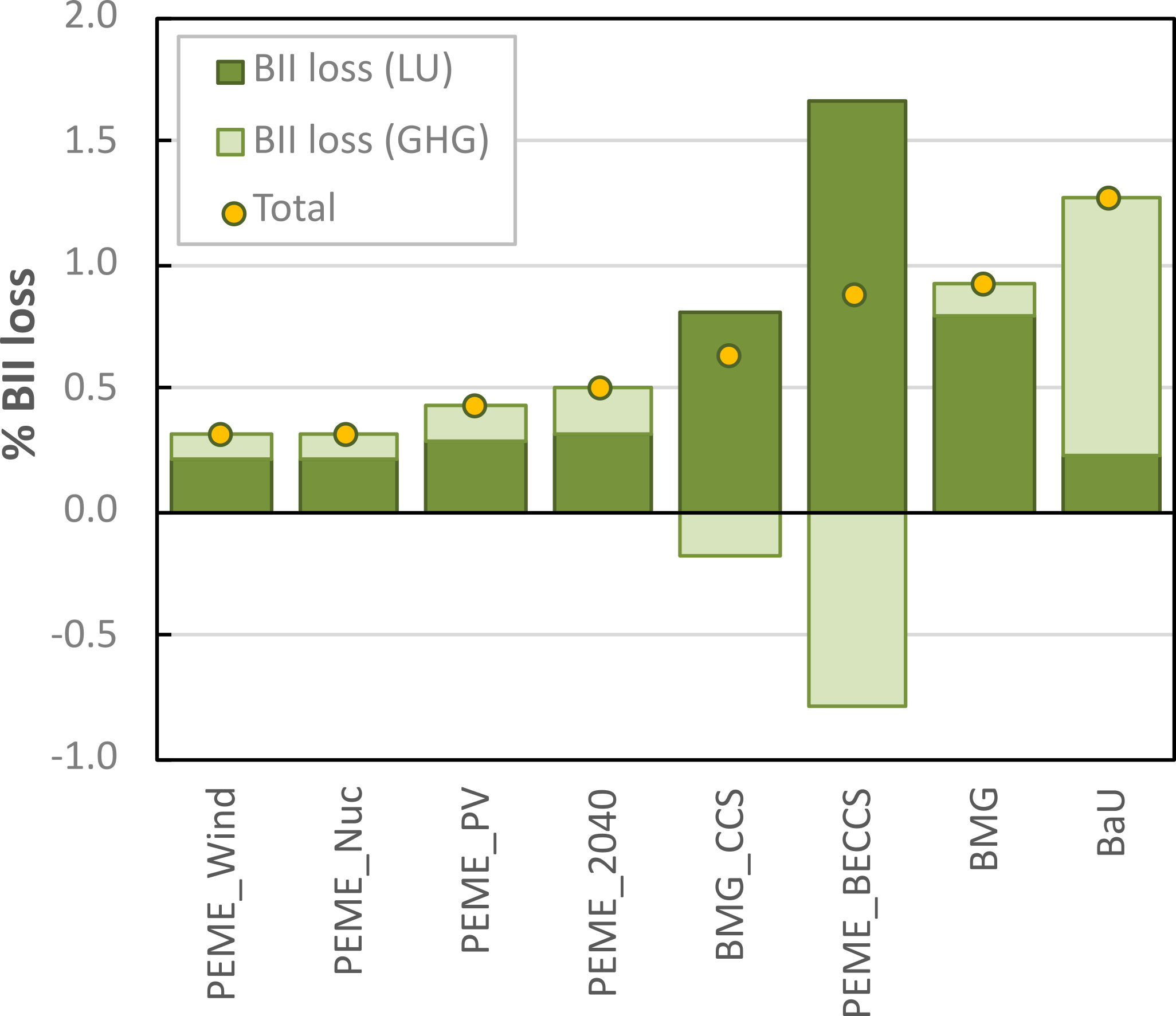

Supplement: SE-005-D1SE00790D-s002 [file SE-005-D1SE00790D-s002.png]
